# Pituitary Apoplexy Presenting as a Transient Ischemic Attack: An Endocrine Emergency

**DOI:** 10.7759/cureus.117859

**Published:** 2026-10-13

**Authors:** Mariana Marques, Joana Couto, Joana Urbano, Pedro Pinto, Iara Fazenda

**Affiliations:** 1 Internal Medicine, Hospital Vila Franca de Xira, Vila Franca de Xira, PRT; 2 Internal Medicine, Unidade Local de Saúde do Alto Minho, Viana do Castelo, PRT

**Keywords:** panhypopituitarism, pituitary apoplexy, pituitary macroadenoma, secondary adrenal insufficiency, transient ischemic attack

## Abstract

Pituitary apoplexy (PA) is an endocrine emergency caused by hemorrhage or infarction of a pituitary adenoma and occurs more frequently in males and patients with macroadenomas. It typically presents acutely with headache, visual disturbances, or impaired consciousness. Focal neurological deficits are uncommon and may be mistaken for a primary cerebrovascular event. Acute secondary adrenal insufficiency is a major contributor to hemodynamic instability and mortality.

A 67-year-old man with arterial hypertension, type 2 diabetes mellitus, dyslipidemia, and chronic obstructive pulmonary disease presented with sudden presyncope, followed by dysarthria and right upper limb weakness. He had a five-month history of headache that had worsened abruptly three days before presentation. On admission, he was febrile (38.5 °C), hypotensive (89/49 mmHg), and tachycardic, with dysarthria, mild right central facial palsy, and grade 4 motor strength in the right upper limb (National Institutes of Health Stroke Scale (NIHSS) score of 3). Computed tomography (CT) angiography revealed an enlarged pituitary gland and 90% calcified stenosis of the left internal carotid artery. The focal neurological deficits resolved completely within approximately 30 minutes of arrival, during fluid resuscitation. However, hypotension persisted despite the administration of 2 L of crystalloid. A 200 mg bolus of hydrocortisone was administered for suspected adrenal insufficiency, resulting in prompt hemodynamic recovery. Baseline cortisol was <1 µg/dL, with an inappropriately low adrenocorticotropic hormone (ACTH) concentration. Magnetic resonance imaging (MRI) confirmed a pituitary macroadenoma with recent hemorrhage, without evidence of infarction on diffusion-weighted imaging. The episode was interpreted as a hemodynamic transient ischemic attack precipitated by PA-associated acute secondary adrenal insufficiency. Antiplatelet therapy, initiated for a presumed primary cerebrovascular event, was discontinued, and the patient was managed conservatively. Follow-up confirmed persistent panhypopituitarism, including central diabetes insipidus, requiring long-term hormone replacement.

PA may mimic a cerebrovascular event through direct vascular compromise or, as in this case, hypotension superimposed on pre-existing carotid artery disease. Empirical hydrocortisone treatment should not be delayed pending laboratory confirmation of adrenal insufficiency. Early MRI is essential to establish the diagnosis and guide management before antithrombotic or endovascular treatment is initiated when a sellar abnormality is identified.

## Introduction

Pituitary apoplexy (PA) is an acute, potentially life-threatening clinical syndrome caused by hemorrhage or infarction of the pituitary gland, usually within a pre-existing, often unrecognized adenoma [1-3]. It occurs in approximately 2%-12% of pituitary adenomas, predominantly non-functioning macroadenomas, and represents the first manifestation of the tumor in up to 80% of patients [1,4,5]. The classical presentation includes sudden-onset headache, visual disturbances, ophthalmoplegia, and varying degrees of impaired consciousness [1,4].

Focal neurological deficits are uncommon but well recognized. Cerebral ischemia may result from mechanical compression or vasospasm of the internal carotid artery (ICA) [6,7] or, less frequently, from systemic hypotension superimposed on pre-existing carotid stenosis [8]. In these cases, the presentation may be mistaken for primary cerebrovascular disease, and the underlying sellar lesion may be overlooked, particularly because non-contrast computed tomography (CT) detects PA in only a minority of cases [1,9].

Endocrine dysfunction is a major determinant of early mortality. Corticotroph deficiency, which occurs in approximately 70% of patients, is a principal cause of hemodynamic instability and hyponatremia [1,3,4]. The resulting hypotension may be refractory to fluid resuscitation and vasopressors but typically responds promptly to glucocorticoid administration [4,10]. Accordingly, current guidance recommends administering empirical parenteral hydrocortisone immediately after baseline cortisol and adrenocorticotropic hormone (ACTH) samples have been collected, without waiting for the results [4,10].

We report the case of a 67-year-old man in whom PA complicated by acute secondary adrenal insufficiency precipitated a transient ischemic attack in the setting of pre-existing calcified left ICA stenosis. This case illustrates how an endocrine emergency may mimic a primary cerebrovascular event and highlights the potential risks of initiating antithrombotic or endovascular treatment before an underlying sellar hemorrhage is recognized.

## Case presentation

A 67-year-old man who was independent in his activities of daily living and professionally active as a metalworker presented to the emergency department with acute dysarthria and right upper limb weakness. His medical history included arterial hypertension, type 2 diabetes mellitus, dyslipidemia, obesity, chronic obstructive pulmonary disease, and chronic alcohol consumption. His regular medications comprised an angiotensin-converting enzyme inhibitor combined with a calcium-channel blocker, bisoprolol, metformin plus empagliflozin, atorvastatin, ezetimibe, allopurinol, and inhaled triple bronchodilator therapy. The angiotensin-converting enzyme inhibitor and calcium-channel blocker had been discontinued during the preceding week because of headache and lower home blood pressure readings, without associated syncope.

He reported a five-month history of persistent headache. Four days before admission, he developed a cough with mucopurulent sputum. Three days before admission, his headache abruptly intensified into a sudden, severe, predominantly left-sided holocranial headache, prompting him to seek emergency care. Cranial CT performed at that time revealed basal ganglia and subcortical calcifications, as well as a left frontal sulcal cortical hyperdensity of uncertain significance, potentially representing calcification or subarachnoid hemorrhage. Following consultation with the regional neurosurgical center, he was discharged with simple analgesia pending reassessment. On the morning of admission, he experienced sudden presyncope followed by dysarthria and right upper limb weakness. He presented to the emergency department approximately one hour after symptom onset and was admitted to the resuscitation room under the stroke code pathway.

On arrival, he was febrile (38.5 °C), hypotensive (89/49 mmHg), and tachycardic (124 beats per minute), with a peripheral oxygen saturation of 95% on room air. He was alert, with a Glasgow Coma Scale score of 15, had no meningeal signs, and was clinically euvolemic. Neurological examination revealed dysarthria, mild right central facial palsy, and grade 4 motor strength in the right upper limb according to the Medical Research Council scale [11], corresponding to a National Institutes of Health Stroke Scale (NIHSS) score of 3. His pupils were symmetrical and normally reactive, with no diplopia, nystagmus, or ophthalmoparesis. Formal ophthalmological assessment during the first week of admission included visual acuity testing, automated perimetry, pupillary examination, assessment of ocular motility, and fundoscopy. Visual acuity was 10/10 bilaterally; automated perimetry revealed no visual field defects; pupillary responses were normal; there was no oculomotor, trochlear, or abducens nerve palsy; and fundoscopy was normal. The Pituitary Apoplexy Score, which assesses the level of consciousness, visual acuity, visual field defects, and ocular paresis, was 0, with no points assigned in any domain [4].

The laboratory results are summarized in Table 1, which also specifies the timing of each sample relative to hydrocortisone administration. Initial investigations revealed hyponatremia (133 mmol/L) and an elevated C-reactive protein concentration of 4.98 mg/dL. Lactate (1.3 mmol/L), creatinine (0.86 mg/dL), and potassium (4.1 mmol/L) were within the normal ranges. Bicarbonate was 30 mmol/L, and capillary ketones were 0.1 mmol/L. Baseline serum cortisol and ACTH levels, measured using samples collected in the emergency department before glucocorticoid administration, were subsequently reported as <1 µg/dL and 3.53 pg/mL, respectively. The full anterior pituitary hormone panel was collected at the same time.

**Table 1:** Laboratory investigations performed during the admission and at four-month follow-up. Admission hormonal samples were drawn before hydrocortisone administration; follow-up samples are annotated with the replacement therapy in use.

Parameter	Timing	Patient value	Reference range
Sodium, admission (mmol/L)	Admission	133	135-145
Sodium, nadir (mmol/L)	Day 7	122	135-145
Sodium, second week (mmol/L)	Second week	142	135-145
Creatinine (mg/dL)	Admission	0.86	0.70-1.20
Potassium (mmol/L)	Admission	4.1	3.5-5.1
Bicarbonate (mmol/L)	Admission	30	22-29
Lactate (mmol/L)	Admission	1.3	0.5-2.2
Capillary ketones (mmol/L)	Admission	0.1	<0.6
Hemoglobin (g/dL)	Admission	16.3	13.0-18.0
White cell count (×10⁹/L)	Admission	10.09	4.0-11.0
Platelets (×10⁹/L)	Admission	147	150-400
Glucose (mg/dL)	Admission	136	70-110
C-reactive protein (mg/dL)	Admission	4.98	<0.5
Blood cultures	Admission	No growth	No growth
Urinary antigen testing	Admission	Negative	Negative
Respiratory viral panel	Admission	Negative	Negative
Serum cortisol (µg/dL)	Admission, before hydrocortisone	<1	6.2-19.4
Adrenocorticotropic hormone (pg/mL)	Admission, before hydrocortisone	3.53	7.2-63.3
Adrenocorticotropic hormone (pg/mL)	Four months, on prednisolone	9.74	7.2-63.3
Thyroid-stimulating hormone (µIU/mL)	Admission, before hydrocortisone	0.04	0.35-4.94
Thyroid-stimulating hormone (µIU/mL)	Four months, on levothyroxine	2	0.35-4.94
Free thyroxine (ng/dL)	Admission, before hydrocortisone	0.64	0.70-1.48
Free thyroxine (ng/dL)	Four months, on levothyroxine	1.03	0.70-1.48
Insulin-like growth factor 1 (ng/mL)	Admission, before hydrocortisone	48.4	49.6-229
Insulin-like growth factor 1 (ng/mL)	Four months	63.4	49.6-229
Prolactin (ng/mL)	Admission, before hydrocortisone	1	2.58-18.12
Prolactin (ng/mL)	Four months	1	2.58-18.12
Luteinizing hormone (mIU/mL)	Admission, before hydrocortisone	0.13	1.7-8.6
Follicle-stimulating hormone (mIU/mL)	Admission, before hydrocortisone	0.7	1.5-12.4
Follicle-stimulating hormone (mIU/mL)	Four months	2.6	1.5-12.4
Total testosterone (ng/mL)	Admission, before hydrocortisone	<0.13	1.27-10.20
Total testosterone (ng/mL)	Four months, before testosterone replacement	0.08	1.27-10.20

Cranial and cervical CT angiography (Figure 1) revealed an enlarged, globose pituitary gland with possible suprasellar extension and a calcified atherosclerotic plaque causing 90% stenosis at the origin of the left internal carotid artery. The left frontal cortical hyperdensity identified three days earlier was again observed and attributed to calcification, with no evidence of subarachnoid hemorrhage. The event was initially interpreted as a large-artery transient ischemic attack, prompting the administration of loading doses of aspirin (500 mg) and ticagrelor (180 mg) in preparation for early carotid stenting. Intravenous thrombolysis was withheld because the neurological deficits were minor, non-disabling, and rapidly resolved.

**Figure 1:**
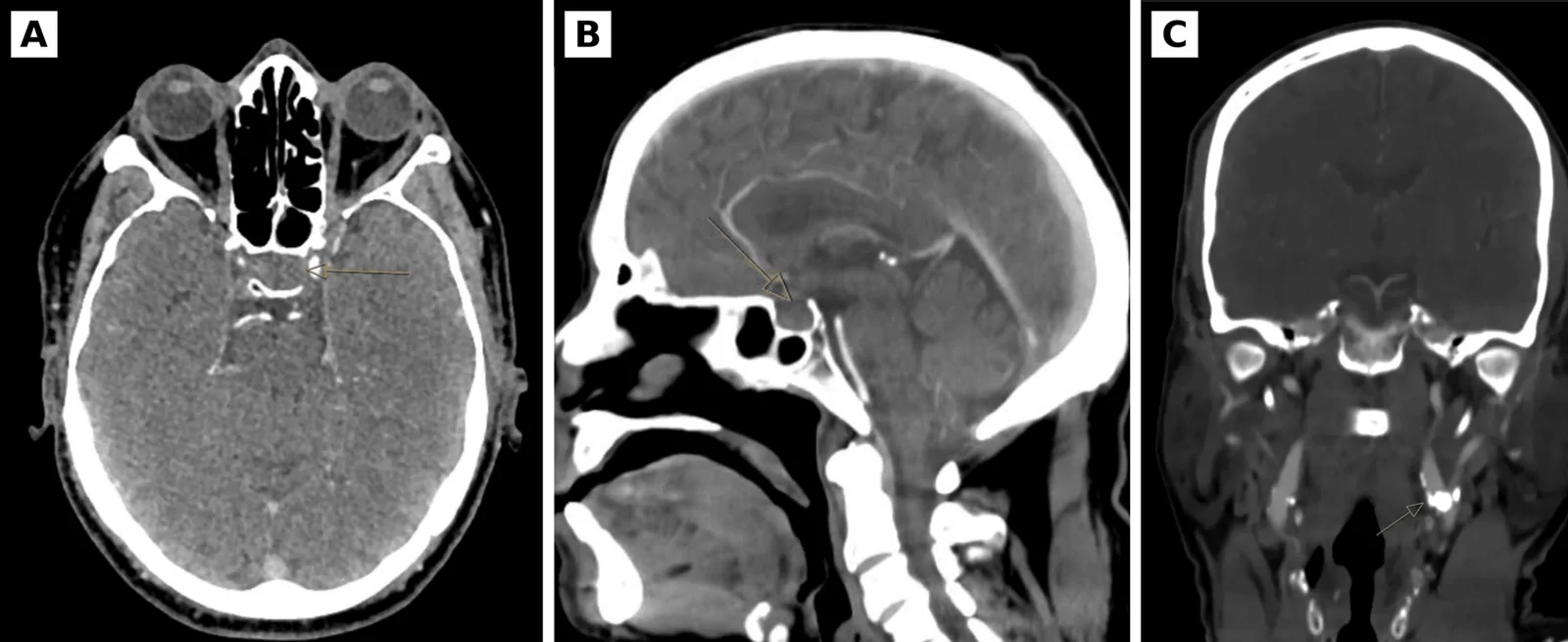
Cranial and cervical computed tomography angiography on admission. (A) Contrast-enhanced axial image at the level of the suprasellar cistern, showing soft-tissue density expanding the sellar and suprasellar region to the left of the midline (arrow). (B) Midline sagittal reconstruction, showing the enlarged, globose sellar contents extending superiorly towards the suprasellar cistern (arrow). (C) Coronal reconstruction showing a calcified atherosclerotic plaque at the origin of the left internal carotid artery, causing 90% stenosis (arrow).

Intravenous crystalloid resuscitation was initiated on arrival. The focal neurological deficits resolved completely within approximately 30 minutes of arrival in the resuscitation room, before glucocorticoid administration, and did not recur. However, hypotension persisted despite the administration of 2 L of crystalloid. Bisoprolol was withheld on admission, whereas empagliflozin was continued; there was no evidence of ketoacidosis. Given the persistent hypotension and hyponatremia, together with fever, headache, and pituitary enlargement, acute adrenal insufficiency with impending shock was suspected. A 200 mg intravenous bolus of hydrocortisone was administered, and vasopressor support was not required. Blood pressure improved promptly to 111/58 mmHg. Fever was documented only on admission, and the patient remained apyrexial thereafter.

Given the fever and recent cough with mucopurulent sputum in a patient with chronic obstructive pulmonary disease, acute tracheobronchitis was provisionally diagnosed. Empirical amoxicillin-clavulanate was initiated in the emergency department and continued for seven days. Chest radiography demonstrated perihilar bronchovascular prominence without consolidation. A septic screen, including blood cultures, urinary antigen testing, and a respiratory viral panel, yielded no significant findings (Table 1). Lumbar puncture was not performed because there were no meningeal signs, the focal neurological deficits had resolved in the emergency department, and alternative explanations were available for both the fever and the neurological presentation. The subsequent clinical course, characterized by no recurrent fever or neurological deterioration despite antibiotic therapy that does not provide adequate coverage for bacterial meningitis, further reduced the likelihood of this diagnosis.

Magnetic resonance imaging (MRI), performed on the same day and reviewed in consultation with the reference neuroradiology center, confirmed a pituitary macroadenoma measuring 14.8 × 12.5 × 12.0 mm with recent intratumoral hemorrhage (Figure 2). There was no cavernous sinus invasion (Knosp grade 0). Diffusion-weighted imaging and apparent diffusion coefficient maps showed no areas of restricted diffusion (Figure 3), supporting a transient ischemic event without established infarction. An incidental cavernoma in the left centrum semiovale, without evidence of recent hemorrhage, was also identified. Given the confirmed pituitary hemorrhage, antiplatelet therapy was discontinued, and carotid revascularization was deferred. The patient was admitted to an intermediate care unit and continued on intravenous hydrocortisone at 100 mg every eight hours.

**Figure 2:**
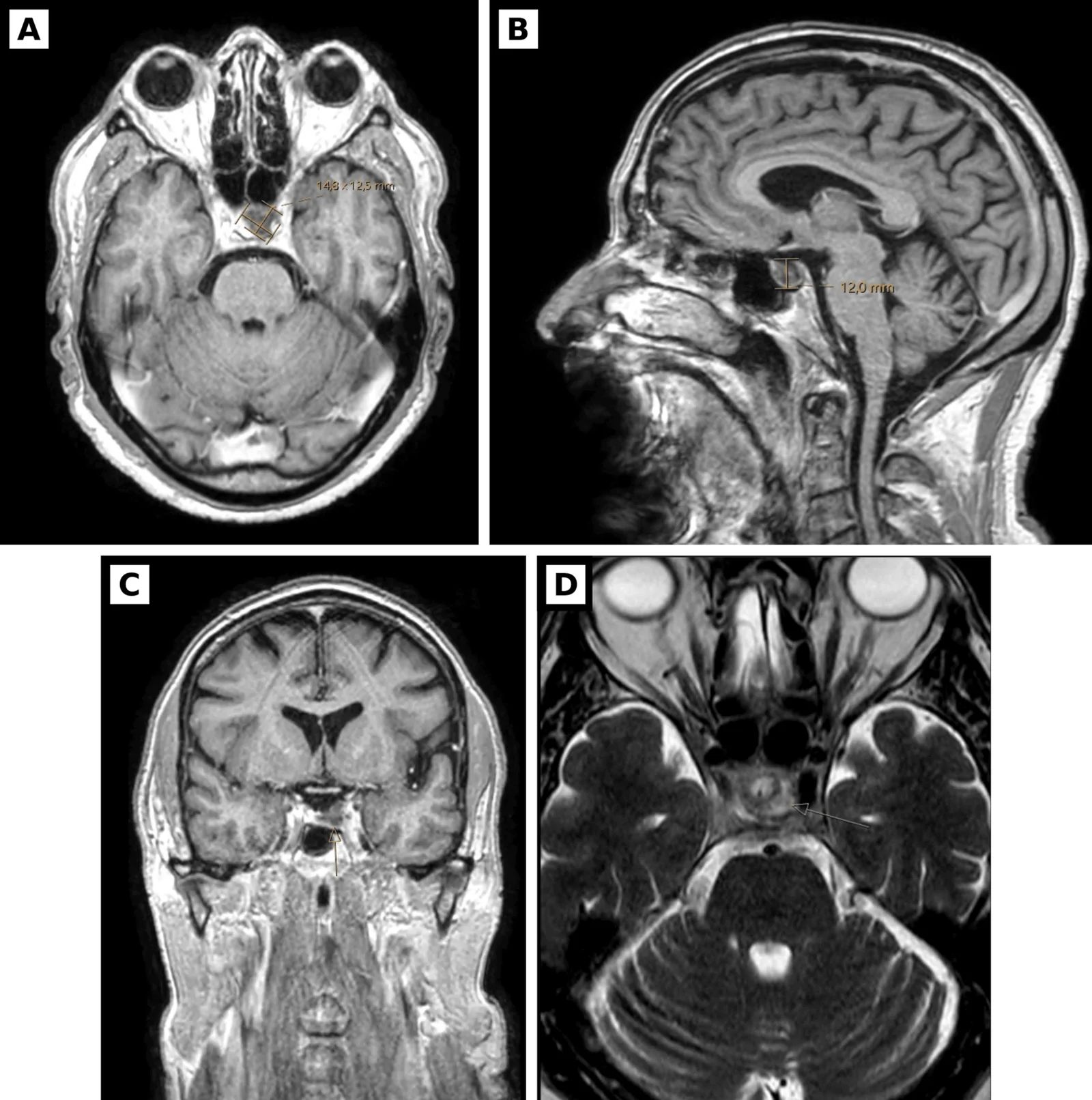
Magnetic resonance imaging of the sellar region. (A) Axial contrast-enhanced T1-weighted image showing a sellar lesion with maximum axial dimensions of 14.8 × 12.5 mm. (B) Sagittal T1-weighted image showing a craniocaudal dimension of 12.0 mm. (C) Coronal contrast-enhanced T1-weighted image showing the lesion occupying an expanded sella (arrow). (D) Axial T2-weighted image showing heterogeneous signal, with a central focus of high signal and areas of lower signal peripherally (arrow), consistent with recent intratumoral hemorrhage.

**Figure 3:**
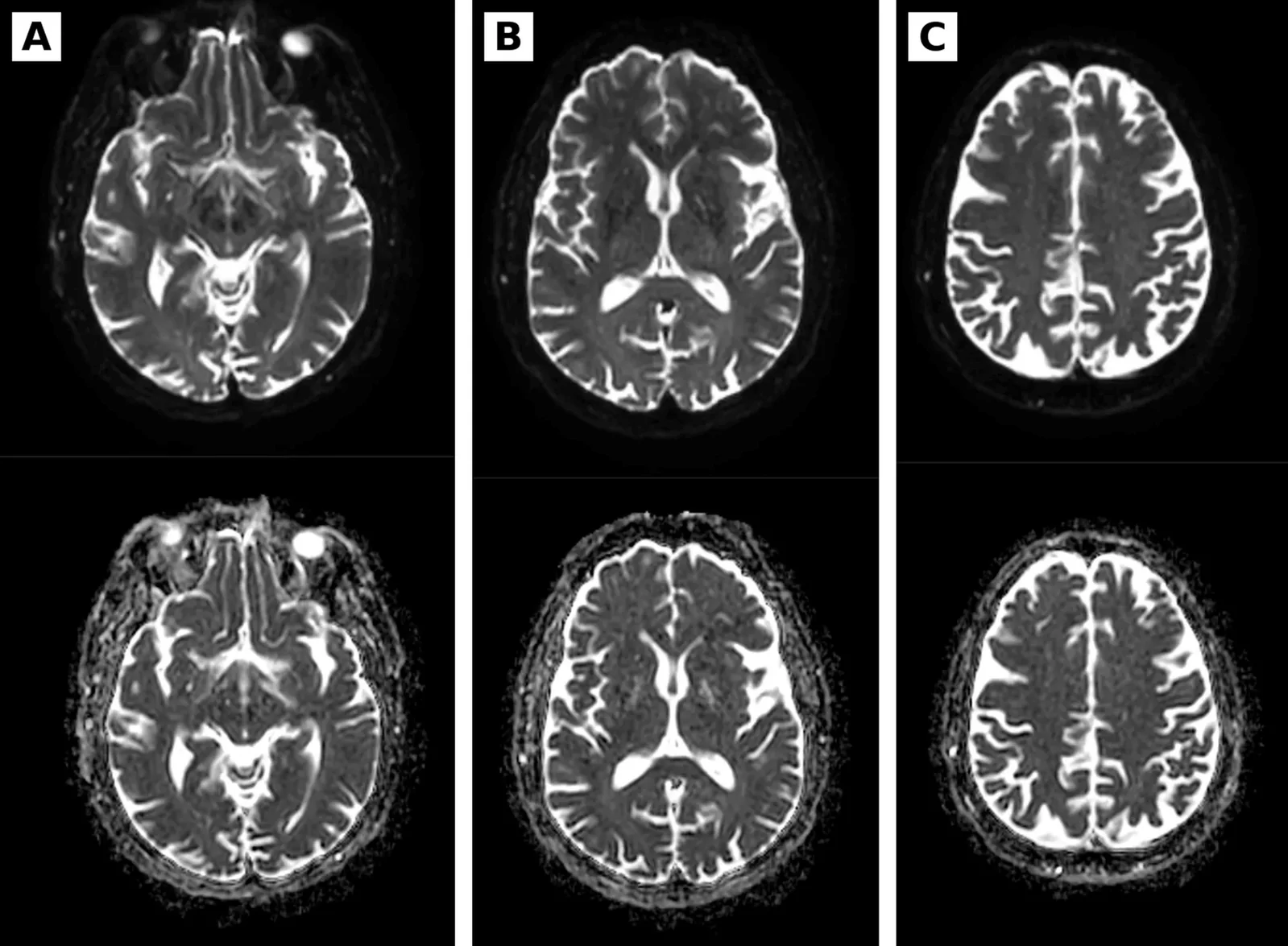
Diffusion-weighted magnetic resonance imaging. Axial diffusion-weighted images (upper row) and corresponding apparent diffusion coefficient maps (lower row) at the level of the midbrain (A), the basal ganglia (B), and the centrum semiovale (C), showing no areas of restricted diffusion and no evidence of acute infarction in the left internal carotid artery territory.

Endocrine evaluation (Table 1) confirmed secondary adrenal insufficiency, characterized by an undetectable baseline cortisol concentration and an inappropriately low ACTH level. Additional anterior pituitary hormone deficiencies were identified: thyroid-stimulating hormone was suppressed (0.04 µIU/mL), with a low free thyroxine concentration (0.64 ng/dL); prolactin was low; gonadotrophin and testosterone concentrations were reduced; and insulin-like growth factor 1 was marginally below the reference range. All hormonal samples were collected on admission, before hydrocortisone administration. However, because the samples were obtained during acute illness, the thyrotroph, gonadotroph, and somatotroph findings may have been partly influenced by non-thyroidal illness and functional hypogonadism and therefore required confirmation. Serum sodium reached a nadir of 122 mmol/L on day 7 and subsequently increased gradually, at a rate of less than 8 mmol/L per 24 hours, reaching 142 mmol/L by the second week. Levothyroxine 75 µg daily was initiated once glucocorticoid replacement had been established.

The case was discussed with the regional multidisciplinary pituitary group. Given the absence of visual compromise or ophthalmoplegia and the favorable clinical course, conservative management was adopted. Hydrocortisone was tapered stepwise to 50 mg every eight hours and then to 50 mg every 12 hours before being replaced with an oral regimen of 30 mg on waking and 20 mg in the afternoon. This regimen was subsequently replaced by prednisolone 5 mg daily, with interval pituitary imaging and endocrinology follow-up arranged. The focal neurological deficits were attributed to hemodynamic cerebral ischemia in the territory of the stenotic left ICA, precipitated by hypotension secondary to acute adrenal insufficiency. Carotid revascularization was deferred, and repeat CT angiography was planned for one month to allow multidisciplinary reassessment after stabilization of the pituitary hemorrhage. At the time of writing, the patient remained clinically stable, apyrexial, normotensive, and free of neurological deficits. Reassessment four months after hospitalization (Table 1) confirmed persistent panhypopituitarism, with corticotroph, thyrotroph, gonadotroph, and lactotroph deficiencies. Total testosterone was 0.08 ng/mL, with an inappropriately normal follicle-stimulating hormone concentration of 2.6 mIU/mL, while prolactin was 1.0 ng/mL. Central diabetes insipidus was diagnosed three months after the acute event. The patient is currently receiving prednisolone 5 mg daily, levothyroxine 100 µg daily, and desmopressin 0.1 mg daily. Intramuscular testosterone at a dose of 125 mg every four weeks was recently initiated.

## Discussion

This case illustrates the key diagnostic challenges of PA in the emergency department. The patient presented with a constellation of findings that could readily lead to misdiagnosis: acute focal neurological deficits suggestive of a primary cerebrovascular event, fever and elevated inflammatory markers suggestive of infection, and hypotension with hyponatremia raising concern for sepsis or a primary cardiovascular process. The unifying diagnosis of hemorrhage into a previously unsuspected pituitary macroadenoma, accompanied by acute corticotroph failure, emerged from the integration of clinical, biochemical, and radiological findings. Importantly, empirical glucocorticoid therapy was initiated before diagnostic confirmation, in accordance with the principle that treatment should not be delayed while awaiting the results of further investigations [1,4].

The cerebrovascular presentation warrants particular attention. Although focal neurological deficits are uncommon in PA, they are well documented and typically arise from mechanical compression or vasospasm of the ICA [6,7]. This case illustrates a less frequently described mechanism: hemodynamic cerebral ischemia resulting from abrupt hypotension due to acute adrenal insufficiency superimposed on severe (90%) calcified stenosis of the left ICA, leading to transient hypoperfusion in the corresponding vascular territory. A similar mechanism has been reported in a patient with tandem carotid stenosis who developed cerebral infarction only after severe hypotension [8]. Three findings support this interpretation: the deficits were consistent with the territory supplied by the stenotic artery; they resolved within approximately 30 minutes during fluid resuscitation, before hydrocortisone administration; and diffusion-weighted imaging showed no evidence of established infarction. Taken together, these findings support the interpretation of the event as a genuine transient ischemic attack precipitated by an endocrine emergency rather than a stroke mimic.

The initial interpretation of the event as a primary large-artery transient ischemic attack led to dual antiplatelet loading and a plan for early carotid stenting. In the presence of an unrecognized recent pituitary hemorrhage, these interventions posed a potential risk of rebleeding and were discontinued once MRI clarified the diagnosis. This sequence illustrates a recognized limitation of the emergency diagnostic pathway: non-contrast CT detects PA in only a minority of cases, and the sellar region may be overlooked when attention is focused on a cortical or carotid abnormality. By contrast, MRI identifies the lesion in the great majority of patients [1,9]. In retrospect, the abrupt worsening of the headache three days before admission may have marked the onset of PA.

Endocrine dysfunction was central to both the physiological disturbance and the subsequent clinical recovery. Corticotroph insufficiency is a major contributor to hemodynamic collapse, hyponatremia, and early mortality in PA [1,3,4]. In this patient, hypotension persisted despite fluid resuscitation but improved promptly after intravenous hydrocortisone administration. Although this response was consistent with adrenal insufficiency, it was not diagnostically specific, as hemodynamic improvement following glucocorticoid administration can also occur in septic shock. The diagnosis therefore relied on the baseline cortisol and ACTH concentrations obtained before treatment. Both UK guidelines and the Society for Endocrinology's Endocrine Emergency Guidance recommend empirical parenteral hydrocortisone when PA with adrenal insufficiency is suspected, administered immediately after baseline cortisol and ACTH samples have been collected, without waiting for the results [4,10]. This sequence was followed in the present case: baseline testing revealed an undetectable cortisol concentration and an inappropriately low ACTH level, supporting secondary adrenal insufficiency. Additional anterior pituitary hormone deficiencies were identified during the acute illness, although these findings required cautious interpretation because of the potential influence of acute physiological stress. Reassessment four months later confirmed persistent panhypopituitarism. Central diabetes insipidus, a less common consequence of PA, was also documented. Posterior pituitary dysfunction may remain clinically masked during glucocorticoid deficiency and become apparent only after glucocorticoid replacement has been initiated [1,4].

Other potential contributors to hypotension were considered. The patient was receiving a sodium-glucose co-transporter 2 (SGLT2) inhibitor, which may contribute to volume depletion, as well as antihypertensive therapy. However, the angiotensin-converting enzyme inhibitor and calcium-channel blocker had been discontinued during the preceding week, and bisoprolol was withheld on admission. Lactate was normal, and the patient was clinically euvolemic, with no evidence of ketoacidosis despite continued empagliflozin (bicarbonate, 30 mmol/L; capillary ketones, 0.1 mmol/L). Furthermore, hypotension persisted despite fluid resuscitation but improved promptly after hydrocortisone administration. Together with the undetectable baseline cortisol concentration, these findings support adrenal insufficiency as the principal cause of hypotension. In retrospect, the lower home blood pressure readings recorded during the preceding week may have reflected evolving corticotroph deficiency.

Fever and hyponatremia also raised the possibility of an infectious etiology. Hyponatremia in PA is multifactorial, arising primarily from impaired free-water excretion due to glucocorticoid deficiency, with a possible contribution from inappropriate antidiuresis [1,4,12]. In this patient, serum sodium reached a nadir of 122 mmol/L on day 7 despite glucocorticoid replacement and subsequently corrected gradually. Persistent hyponatremia may have reflected an additional contribution from inappropriate antidiuresis. Fever, which was documented only on admission and did not recur after hydrocortisone administration, may have been attributable to adrenal crisis, the concomitant respiratory infection, or both. Hypothalamic involvement is a less likely explanation given the absence of persistent pyrexia. Whether the respiratory infection or pre-existing arterial hypertension contributed to the onset of PA remains uncertain. A discernible precipitating factor is not identified in a substantial proportion of published cases [13].

The order of hormone replacement also warrants emphasis. Glucocorticoid replacement must be established before levothyroxine is initiated, as thyroid hormone increases cortisol clearance and may precipitate an adrenal crisis in patients with untreated adrenal insufficiency [4,12]. This sequence was followed in the present case.

The definitive management of PA remains guided by clinical judgment, as randomized controlled trial evidence is lacking. Observational studies suggest that, in patients without severe or progressive neuro-ophthalmological deficits, conservative management can achieve visual and endocrine outcomes comparable to those of surgery [9,14-16]. Current guidelines recommend urgent surgical decompression for patients with severely reduced visual acuity, severe and persistent visual field defects, or deteriorating consciousness. The Pituitary Apoplexy Score has also been proposed to help standardize clinical assessment [4]. In the present case, the patient had normal visual acuity and visual fields, no ophthalmoplegia, and a Pituitary Apoplexy Score of 0. The multidisciplinary pituitary team therefore opted for conservative management with interval imaging, in accordance with published guidance [4,10]. The coexisting symptomatic carotid stenosis added further complexity, as revascularization would require antiplatelet therapy, which could increase the risk of further bleeding in the setting of recent pituitary hemorrhage. A staged, individualized approach was therefore agreed upon, with carotid intervention deferred until the patient had stabilized and the sellar lesion could be reassessed.

This report has the limitations inherent in a single-case observation. The diagnosis was based on clinical, biochemical, and radiological findings without histopathological confirmation, as conservative management was pursued. The attribution of the focal neurological deficits to hemodynamic ischemia remains presumptive, although it is supported by their temporal association with hypotension, their distribution within the territory of the stenotic artery, and the absence of infarction on diffusion-weighted imaging. The initial thyrotroph, gonadotroph, and somatotroph results were obtained during acute illness. At four months, thyrotroph and gonadotroph deficiencies were confirmed, whereas insulin-like growth factor 1 was within the reference range. Formal ophthalmological assessment was performed during the first week of admission rather than within the first 24 hours; however, the patient reported no visual symptoms and had no visual or oculomotor abnormalities on the initial examination. Despite these limitations, the combination of acute headache, MRI evidence of a macroadenoma with recent hemorrhage, and documented acute corticotroph deficiency strongly supports the diagnosis of PA [1,4].

## Conclusions

PA is a rare but genuine endocrine emergency that may present with headache, focal neurological deficits, fever, hyponatremia, and hypotension, mimicking primary cerebrovascular disease, meningitis, or sepsis. This case illustrates how hypotension due to acute adrenal insufficiency may precipitate cerebral ischemia in a patient with pre-existing carotid stenosis and highlights two practical lessons for emergency care. First, persistent hypotension despite fluid resuscitation in a patient with acute headache or a sellar abnormality should prompt immediate empirical parenteral hydrocortisone after baseline cortisol and ACTH samples have been collected, without delaying treatment while awaiting the results. Second, early MRI should be considered when imaging reveals a sellar abnormality, as antithrombotic treatment or endovascular intervention for a presumed primary cerebrovascular event may increase the risk of complications when pituitary hemorrhage is present but unrecognized. Early recognition, prompt glucocorticoid replacement, and multidisciplinary management are central to the management of this potentially fatal condition.
